# A longitudinal study of disordered eating in Australian adolescents: modelling psychosocial and individual risk factors

**DOI:** 10.1186/2050-2974-2-S1-P1

**Published:** 2014-11-24

**Authors:** Emma Jarosz, Isabel Krug, Primrose Letcher, Craig Olsson

**Affiliations:** 1 The University of Melbourne, Melbourne, Australia; 2 School of Psychology, Deakin University, Melbourne, Australia; 3 Murdoch Children’s Research Institute, Melbourne, Australia; 4 The University of Melbourne (Paediatrics and Psychological Sciences), Melbourne, Australia; 5 The Royal Children's Hospital, Melbourne, Australia

## Objective

To test whether a sociocultural pathway model was predictive of a) disordered eating (DE) behaviour concurrently (Time 1[T1];age 12-13) and b) DE behaviour longitudinally (Time 2[T2];age 15-16). A further aim was to assess whether the risk factors included in the final model were moderated by negative emotionality.

## Method

Participants included 508 adolescent girls assessed through the Australian Temperament Project (ATP). Predictor variables comprised sociocultural pressure, thin-ideal internalisation, negative comparisons and body dissatisfaction. DE was assessed through the EDI-2 subscales drive for thinness and bulimia.

## Results

The model fit for the final Structural Equation Model (SEM) provided an acceptable fit, [2(28)=105.88,p<.001,RMSEA,= .07,CFI=.95, SRMR =.05]. Path analysis revealed sociocultural pressures to diet increased concurrent body dissatisfaction (=.70;p<0.05) and DE at T1 (=.56;p<.05). Internalisation of the thin-ideal and negative appearance comparisons partially mediated the effect of sociocultural pressures on body dissatisfaction (=.06,p<.05). There was however still a significant direct path between sociocultural pressure and body dissatisfaction. Prospectively, none of these risk factors predicted later adolescent DE at T2. Moderation analyses revealed that negative emotionality did not moderate the effect of any of the risk factors assessed in the SEM.

## Conclusions

The results highlight the importance of longitudinal and multiple risk factor research for informing the development of prevention programs for DEs.

